# Extirpation of a Large Part of the Superior Maxillary Bone, on Account of a Schirrous Tumor Developed in the Antrum

**Published:** 1844-03

**Authors:** F. G. Del Valle


Art. III.—*Extirpation of a large part of the Superior Maxillary Bone, on account of a Schirrous Tumour developed in the Antrum.* By Dr. F. G. del Valle. Translated from the Spanish by the Junior Editor.*

Seraphina N., negress, 18 years old, field-laborer, of common stature, lymphatico-nervous temperament, entered the Hospital of St. Francis, in Havana, towards the end of August, 1842, with a tumor occupying all the left side of the face, covered by the common integuments which were much distended but healthy. The tumor was as large as a common orange, filled the whole mouth, and viewed from the side, projected forwards an inch beyond the upper lip. Anteriorly it was about three inches in diameter, with a cor-

* We are indebted to the *Repertorio Medico-Habanero* for this article. C.

responding circumference, which was ulcerated and contained three incisor teeth. The tumor extended along the alveolar process, as far as the right canine tooth, and backwards to the posterior part of the palatal arch on the left side, giving room only for the last two molar teeth, which were firm and healthy, and leaving free the palate bone of the same side, and a small portion of the maxillary with which it articulates. Superiorly, it rested below the cheek-bone (*pómulo*), and four lines below the orbit, in the direction of the nose, projecting outwards in an extraordinary manner, and turning upwards and to the right, giving a most disagreeable and frightful aspect to the countenance. The tumor was hard, one part of it a deep red color, the other of the natural color of the mucous membrane of the mouth; it was covered with a multitude of tortuous vessels in a dilated and varicose state, it did not yield upon pressure, the pain was not very severe, and nothing issued from the ulcerated portion. The tonsils and cervical glands remained healthy, as well as the tongue and adjacent parts; mastication was very difficult and speech almost unintelligible.

An inquiry into all the circumstances connected with the appearance and development of the disease, showed only that two years previously the patient had received a kick from an ox on the side of the face, which no doubt fractured the superior maxillary bone—no other cause could be discovered. In view of the time that had elapsed, the symptoms present, and the visible organic lesion, I did not hesitate to class it as a schirrous tumor and probably an osteo-sarcoma of the maxillary sinus. The only remedy, therefore, was extirpation, which would involve the greater portion of the maxillary bone, and endanger the individual's life. A consultation was held, at which were present Professors Gutierrez, Castro, Jorrin, Bodman, Pinelo, and A. Valdes. Various reflections were made concerning the nature of the tumor; some suspected it to be aneurismatic fungus, and proposed tying the carotid previous to the operation. A majority decided that this was unnecessary, since there would be no difficulty in controlling the hemorrhage during the operation; all agreed as to the absolute necessity of extirpation, without which the death of the patient was inevitable.

On the appointed day, everything being in readiness, I proceeded to remove the tumor, in the presence of a number of Professors and students of the school of medicine. The patient being placed on a firm seat, with the necessary light, the arms and legs bound for greater security, and the head resting on the breast of an assistant, I began by making a curved incision which divided the integuments and muscles of the face, from a point in the zygomatic arch, an inch from the outer angle of the eye, to the commissure of the lips; three arteries presented themselves, and were secured by Dr. Gutierrez. I then dissected up the cheek as far as the orbit, and anteriorly, in order to display the tumor; lifted the nose upwards, separating it from the alveolar border; applied the saw horizontally to the inferior part of the malar bone; then with a chisel and mallet divided the ascending process of the superior maxillary, at its junction with the nasal and near the ungual bones; next the canine tooth of the right side was extracted, the nasal cartilage and the vomer were cut with sharp scissors, and with the forceps of Liston the alveolar arch was divided at a single stroke as far as the nasal fossae. Afterwards Dr. Gutierrez, with the same forceps applied before the second molar, cut into the maxillary sinus; the palatal arch was then divided by means of a strong curved knife, cutting from within outwards. The tumor being now circumscribed and movable was lifted out with very little force, leaving only many portions of lardaceous nature adhering to the internal and posterior surface of the antrum, and to the left turbinated bones; these portions were all separated by means of the fingers and instruments, during which it became necessary to extract the last two molars which had been suffered to remain, as the alveolar border where they stood was evidently diseased. The arterial hemorrhage was restrained by means of torsion, and lotions of cold water constantly applied, without resorting to the actual cautery which was in readiness. After waiting a moment, for the assistants to assure themselves that the morbid mass was removed and the vessels secured, the soft parts were brought down, and retained by three interrupted and one twisted suture, assisted by strips of adhesive plaster, dry lint internally, cribriform compresses, bandages, &c.; the patient was then carried to bed, and ordered a slightly anodyne draught, with tepid water and sugar as the only aliment.

On the third day there was fever, slight coma, profuse ptyalism, with fetor, and pain of the face: the anodyne was suspended, warm pediluvia and sinapisms were directed, with sweetened water for nourishment. The patient continued in the same state until the fifth day, when diarrhœa supervened, apparently in consequence of the water drank. An opiate was ordered with toast water in small portions every four hours. The dressings were now removed for the first time, and the wound presented a good aspect.

Internally it was washed with barley-water, slightly acidulated, and then dressed with dry lint; externally it was covered with a cribriform compress lightly spread with cerate. On the sixth day the patient was free from fever and headache; on the ninth two of the pins were removed. On the fifteenth the external wound was perfectly united, the three ligatures on the arteries still remaining; the sutures were all removed, and it was dressed with dry lint. The inflammation followed its usual course internally; some portions of the soft parts, contused during the operation, became sphacelated and were removed; tumefaction subsided after exfoliation of small pieces of bone; and finally on the twentieth day, the entire surface was free, and covered with granulations, which required to be touched subsequently with the nitrate of silver. Acidulated barley-water, weak solutions of the sulphate of zinc, nitrate of silver, and lint, generally dry, sometimes covered with cerate, were used until the time of complete cicatrization, which occurred 48 days after the operation.

As the patient is young, and the skin covering the tumor sound, the deformity is very slight, and even internally there is not so great a loss of substance as might be supposed from the enormous mass that was removed. It is true, the inferior turbinated bone, and the entire alveolar process, from the right canine to the last molar tooth, are wanting, leaving above so much only of the left maxilla as contributes to the formation of the orbit; but as there remain below four lines of the bone in its union with the palate bone, she still retains in a healthy condition a third of the arch of the palate; and by covering the hollow which is left with very fine and well compressed lint, can speak though with some difficulty; and the passage of air and food through the nose being prevented, mastication is performed with the four molars of the opposite side.

